# Free-field characterization via directional transmission through a nanoaperture

**DOI:** 10.1186/1556-276X-8-326

**Published:** 2013-07-16

**Authors:** Jussi Rahomäki, Heikki J Hyvärinen, Shakil Rehman, Jari Turunen

**Affiliations:** 1 Department of Physics and Mathematics, University of Eastern Finland, P.O. Box 111, Joensuu FI-80101, Finland; 2 Rocsole Ltd, Microkatu 1, P.O. Box 1188, Kuopio FI-70211, Finland; 3 Department of Bioengineering, National University of Singapore, 7 Engineering Drive 1, Singapore 117574, Singapore; 4 Singapore-MIT Alliance for Research and Technology, 3 Science Drive 2, Singapore 117543, Singapore

**Keywords:** Fourier modal method, Subwavelength aperture, Field probe, Extraordinary optical transmission

## Abstract

We propose a scheme based on extraordinary transmission of light through a single nanoaperture, surrounded by periodic corrugations, for direct characterization of focal-region optical fields with subwavelength-scale structure. We describe the design of the device on the basis of rigorous diffraction theory and fabricate a prototype using a process that involves electron beam lithography, dry etching, and template stripping. First experimental results performed with a transmission-type confocal optical microscope demonstrate the potential of the method.

## Background

The ability of arrays of subwavelength apertures in a metal screen to transmit more light than geometrical considerations suggest has been known in grating theory for several decades (see Sect. 7.3 of [1]). However, the interest in this phenomenon exploded only when Ebbesen et al. [2] coined the term extraordinary optical transmission and suggested the role of surface plasmons in physical explanation of the effect. Subsequent studies have shown, e.g., that single subwavelength apertures in metal screens can also exhibit unexpectedly high transmission, especially if surrounded by corrugations on the entrance surface of the screen. On the other hand, corrugations on the exit surface can give rise to directional emission from the aperture, known as the beaming effect. We refer to [3-6] for a detailed coverage of such effects and their applications in different fields of science and technology.

**Figure 1:**
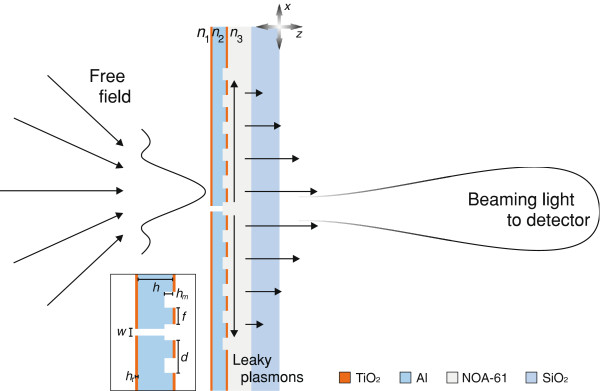
**The system concept. **The concept of the nanoslit-based probe for characterization of subwavelength-structured free fields. The inset shows the design parameters of the device: the aperture width *w*, the screen thickness *h*, and the thickness *h*_*t*_ of a thin TiO_2_ layer as well as the period *d*, groove depth *h*_*m*_, and trench width *f* of the corrugations.

In this paper, we study the possibility of using a single subwavelength aperture, surrounded by periodic corrugations on the exit side of the metal screen, in direct observation of the structure of tightly focused fields in the focal region. The proposed scheme is illustrated in Figure 1, along with the materials and parameters of our demonstration device. The field in the focal region is scanned with a tiny aperture in a finitely conducting metal screen. Surface plasmons are generated on the exit side, which propagate along the surface away from the aperture; these surface-bound waves are coupled by the corrugations into a directional field propagating into a detector in the far zone of the aperture (in practice, using a microscope objective).

In the forthcoming sections, we describe the methods used to design the nanoscale field probe and to fabricate its first prototype. We also give preliminary experimental results on applying the prototype to measure directly the spot size of a tightly focused laser beam. For simplicity of design and fabrication, we employ in this study a slit aperture surrounded by straight-line corrugations, though the final goal is to use a circular aperture surrounded by a grating with concentric circular grooves.

## Methods

**Figure 2:**
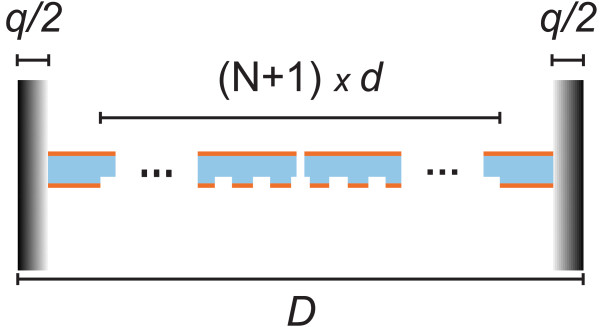
**Computational model. **A schematic illustration of the computational cell with superperiod *D *containing the slit, *N *grooves, and a perfectly matched layer with thickness *q*.

The numerical design of the field probe shown in Figure 1 was performed by the Fourier Modal Method (FMM), which is a standard algorithm for rigorous electromagnetic analysis of diffractive structures [7]. The FMM is directly applicable to periodic structures only, but non-periodic devices such as the one shown in Figure 1 can be treated by adding a perfectly matched layer (PML) between each ‘superperiod’ as shown schematically in Figure 2; the PML acts as an artificial infinite space between the adjacent superperiods [8]. The superperiod (length *D*) contains the slit aperture surrounded on both sides by a finite grating with period *d* and *N*/2 grooves, as well as the PML with thickness *q*.

Since a HeNe laser with wavelength *λ* = 632.8 nm was to be used in the experiments, the refractive indices of the materials were taken in the design to correspond to this wavelength. We used the following values: 1.38 + 7.62i for Al, 2.37 for TiO_2_, 1.56 for the optical adhesive NOA-61, and 1.46 for SiO_2_. The medium on the entrance side was assumed to be either air or water, and the NOA-61 on the exit side could be assumed to extend to infinity because its thickness is several tens of micrometers. The thin TiO_2_ layers (thickness *h*_*t*_ = 10 nm) shown in Figure 1 have no operational functionality but are introduced only to facilitate the fabrication process as described below. The width *w* of the slit was fixed to 50 nm in order to obtain high spatial resolution and to keep the transmitted signal on a reasonable level for the experimental measurements. Hence, the variables left for the FMM-based design are *h*, *h*_*m*_, *d*, and *f*. The choice of these parameters will be discussed in the next section. A TM-polarized cylindrical Gaussian wave with its waist located at the entrance plane of the probe was assumed in the numerical simulations: the non-vanishing magnetic field component was taken to be of the following form:

(1) $$H_{y}(x)=\text{exp}\left(-x^{2}/W^{2}\right),$$

with the value *W* = 200 nm being assumed in all numerical simulations. In the FMM calculations, this field was represented using its sampled angular spectrum of plane waves, as usual, when dealing with incident fields of finite spatial extent.

**Figure 3:**
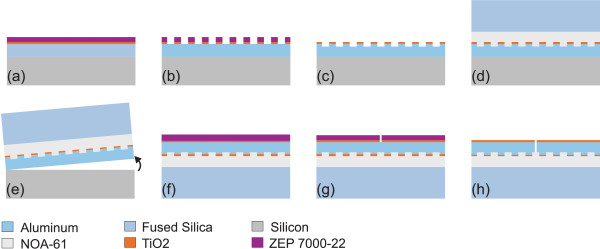
**Process flow.** The fabrication process flow of the device in Figure 1. **(a)** Sample. **(b)** Electron beam patterning of the grooves in resist. **(c)** Dry etching of the corrugations. **(d)** Gluing the SiO_2_ substrate. **(e)** Template stripping. **(f)** Resist coating. **(g)** Patterning of the slit. **(h)** Dry etching of the slit.

Figure 3 shows the fabrication process flow. First a 180-nm-thick aluminum film was deposited by electron beam evaporation (Leybold L560, Oerlikon Leybold Vacuum GmbH, Cologne, Germany) on a 2-in diameter Si (100) wafer. A 10-nm-thick titanium dioxide film was added on top of the aluminum by atomic layer deposition to work as an etching mask and to cover the aluminum film against oxidation. Finally, a 180-nm layer of ZEP 7000-22 electron beam resist was spin-coated on top, resulting in the sample illustrated in Figure 3a. The grating structure was patterned into the resist using electron beam lithography (Vistec EBPG5000+ES HR, Jena, Germany). The resist was used as a mask for TiO_2_ layer etching (Oxford Instruments PlasmaLab 80, Oxfordshire, UK); further, the TiO_2_ layer served as a mask for Al etching (Oxford Instruments PlasmaLab 100). A 2-in, 0.5-mm-thick SiO_2_ wafer was attached on the grating surface using UV-curable glue (Norland Optics, NOA-61). A heat- and solvent-assisted process was used to ensure glue penetration into the narrow grating holes [9]. To achieve appropriate adhesion properties, two nanometers of Al_2_O_3_ was added on the grating before glue. After a 60-min bake in a UV oven, the silicon substrate was detached from the Al surface by template stripping technique [10] using a pressurized N_2_ flow. The process continued on the newly revealed Al surface. Essentially, by repeating the initial steps, a 10-nm layer of TiO_2_ was deposited on the Al surface, followed by coating with a 180-nm ZEP 7000-22 resist layer. An alignment electron beam exposure was applied to write the slit structure, and the final etching steps followed the ones used on grating-side etching. The completed experimental device had an area of 1 mm^2^, with a 1-mm-long slit placed at the center of the device.

**Figure 4:**
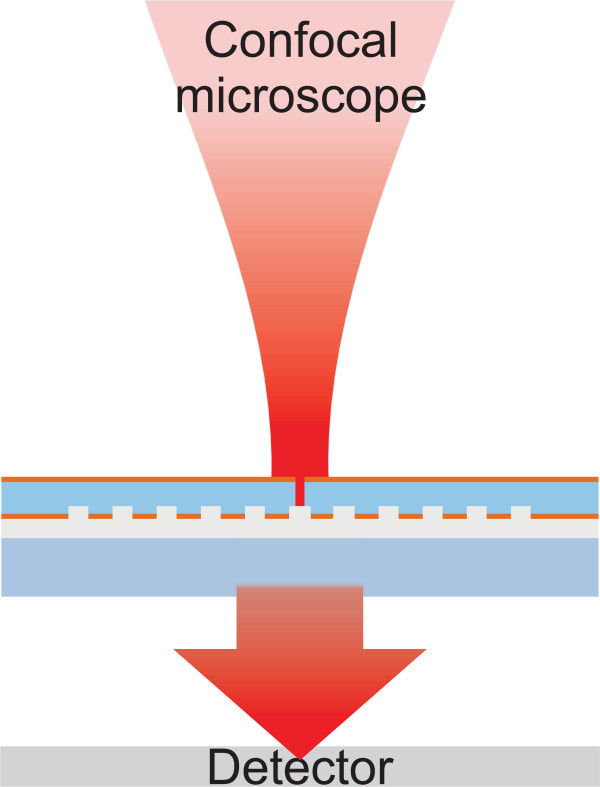
**Measurement configuration. **A schematic configuration for characterization of the field probe, which is placed in the object plane of a scanning confocal transmission microscope. The same geometry is used to measure the profile of the incident field by scanning it across the probe.

The structure was characterized by a scanning electron microscope (LEO 1550 Gemini, Carl Zeiss AG, Oberkochen, Germany) and an atomic force microscope (AFM AutoProbe M5, Veeco Instruments Inc., Plainview, NY, USA). The configuration illustrated schematically in Figure 4 was used both to analyze the transmission properties of the field probe and to test its resolution in the characterization of tightly focused fields. A Gaussian beam (wavelength 632 nm) from a scanning confocal transmission microscope was used to illuminate the slit. The beam was focused through a × 60 microscope objective with a numerical aperture (NA) = 1.2 using water immersion. The transmitted signal was collected by a photomultiplier tube (PMT) detector through an oil-immersion condenser lens with NA = 1.4 (not shown in Figure 4). Since a confocal microscope was used for illumination, the resolution measurements could be performed conveniently by scanning the incident spot perpendicularly across the slit and observing the output of the PMT detector. Such line scans were typically performed over several *y* positions across the slit, which allowed averaging of the resulting (slightly different) intensity signals.

## Results and discussion

The initial optimization of the parameters was performed by looking for optimal plasmon coupling by the corrugations. The starting point for grating period was chosen by matching the real part of the propagation constant *k*_sp_ of the surface plasmon at a smooth metal dielectric interface with a normally exiting plane wave, which gives

(2) $$d=\frac{2\pi }{\left|ℜ\{k_{\text{sp}}\}\right|,}$$

for diffraction orders ±1 of the grating. In our case (Al/NOA interface, *λ* = 632.8 nm) *k*_sp_ ≈ (15.9 + 0.12i) *μ*m^-1^, which gives *d* ≈ 400 nm. Since the effective surface plasmon propagation distance along a non-corrugated surface is only 1/*I*{*k*_sp_} ≈ 21.5*d*, the number of grooves on each side of the slit was set to 9, which should ensure efficient outcoupling of the surface plasmon field. Leaving some space (≈ 4 *μ*m) between the corrugated region and the PMLs as indicated in Figure 2 lead us to choose a superperiod *D* = 20 *μ*m in the FMM design.

**Figure 5:**
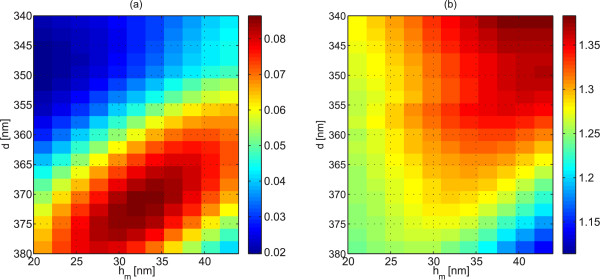
**Corrugation design.** Transmission side corrugation optimization using as the criterion either **(a)** the zeroth-order efficiency or **(b)** the total transmission efficiency, which are plotted here as functions of the corrugation height *h*_*m*_ and period *d*.

It is conceivable that the radiant intensity in the direction normal to the interface (which in the FMM analysis corresponds to the zero-order diffraction efficiency *η*_0_ of the superperiodic grating) may be used as the criterion to optimize the performance of the transmission side corrugations in the present application. Alternatively, one might consider using the integrated radiant intensity in the positive half-space, i.e., the sum *η* of the efficiencies of all transmitted propagating orders in the FMM analysis. The best criterion would in principle be the integrated radiant intensity within the NA of the collection optics, but this would depend on the type of detection scheme used. We therefore compare the first two methods in Figure 5 by plotting in Figure 5a the zeroth-order efficiency *η*_0_ and in Figure 5b the total transmission efficiency *η* for different values of groove depth *h*_*m*_ and grating period *d*, assuming a fill factor *f*/*d* = 0.5. The optimum values of the parameters differ somewhat, with zeroth-order criterion giving a somewhat larger period and a considerably smaller groove depth than the criterion based on total transmission. Although high-numerical-aperture collection optics was used in our experiments, we chose the former criterion, which would allow the use of a detector without any collection optics provided that it covers a reasonable solid angle in the far field. Thus, the grating parameters *d* = 370 and *h*_*m*_ = 30 nm were chosen for further design.

**Figure 6:**
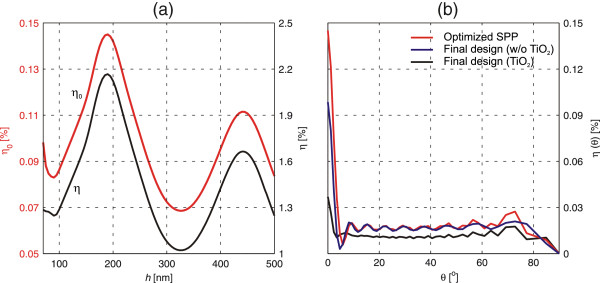
**Transmission efficiency. (a) **Variation of the zero-order efficiency *η*_0 _(red line, left-hand scale) and the total transmission efficiency *η* (black line, right-hand scale) as a function of the Al film thickness *h*. **(b) **Angular dependence of transmission efficiency: surface corrugation optimized for SPP coupling (green line), the final design without (blue line) and with (black line) the TiO_2_ layers.

The final step in the design of the field probe is to choose the optimum thickness *h* of the Al layer. As is well known, if *h* is at approximately 100 nm, a solid Al film is practically opaque at visible wavelengths. Figure 6a illustrates the effect of the film thickness *h* in the transmission of the device consisting of the slit and corrugations as designed above. The results are shown using either *η*_0_ or *η* as the criterion (note the different scales). They exhibit a typical Fabry-Perot-like variation of transmittance through a subwavelength-width metal-insulator-metal waveguide of finite length *h*. With both criteria, the first maximum is obtained at *h* ≈ 180 nm; hence, this value was chosen for further simulations and experiments.

To get an idea of the far-field radiation pattern of the probe, we plot in Figure 6b the angular distribution of transmission efficiency *η*(*θ*), which in FMM calculations means plotting the efficiencies *η*_*m*_ of all propagating orders of the superperiodic grating. Comparison of the green and blue lines illustrates the improvement of transmission achieved by final optimization of the corrugation on the exit face of the Al layer. In the design process, the presence of the thin TiO_2_ layers shown in Figure 1 was ignored. The effect of including these layers in the analysis is illustrated by the black line, and it is seen to reduce *η*_0_ slightly (in principle, the design could be improved slightly by optimization of the parameters in the presence of these layers). In all cases considered, however, the strong and narrow central peak, with half-width at half-maximum of approximately 3°, is surrounded by a wide ‘pedestal’ extending over the entire half-space. Hence, if the light efficiency of the system is a critical factor (which was not the case in our experiments), the use of high-numerical-aperture collection optics is recommended despite of the beaming effect being utilized in the design.

**Figure 7:**
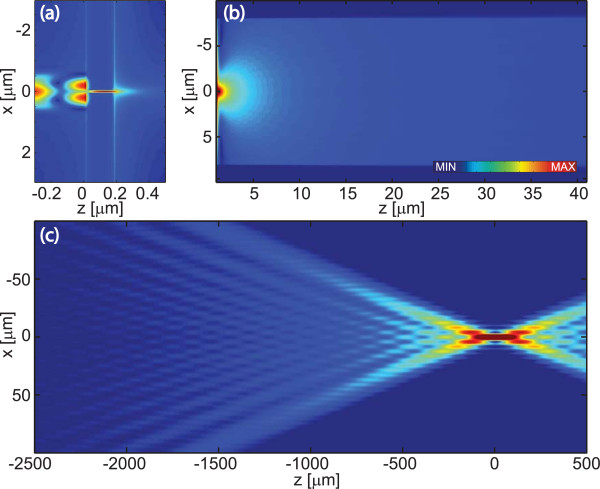
**Simulated diffraction from a slit without corrugations. (a) **The near-field and **(b) **propagated distributions of the magnetic field amplitude |*H*_*y*_| in the neighborhood of a single slit in the Al screen. **(c) **The field propagating towards and past the image plane *z* = 0 in an Abbe configuration with numerical aperture 1.4 and magnification × 10.

**Figure 8:**
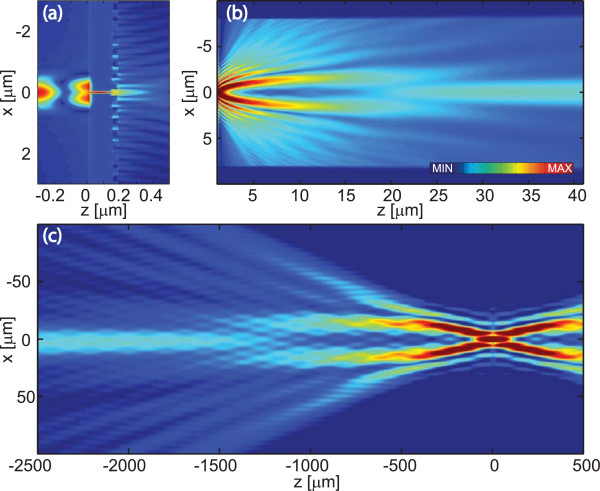
**Simulated diffraction from a slit with corrugations. (a)** The near-field and **(b)** propagated distributions of the magnetic field amplitude |*H*_*y*_| in the neighborhood of a slit surrounded by corrugations. **(c)** The field propagating towards and past the image plane *z* = 0 in an Abbe configuration with numerical aperture 1.4 and magnification × 10. The complete field probe with the slit surrounded by corrugations is considered.

Let us next consider in more detail the advantages gained by adding the corrugations on the rear side of the Al film. Field amplitude distributions |*H*_*y*_(*x*,*z*)| without and with corrugations are compared in Figures 7 and 8. The fields inside the probe and in its wavelength-scale neighborhood are illustrated in Figures 7a and 8a, where the regions 0 ≤ *z* ≤ 0.18 *μ*m contain the Al film. A close inspection of these figures shows the interference of the reflected and the incident fields, the high intensity inside the slit, and the slight penetration inside all the metal surfaces. Also seen are the plasmon waves that propagate away from the slit; these are particularly apparent on the exit side. Figure 8a also illustrates the rapid outcoupling of the plasmons by the first few grooves, compared to the much slower attenuation of the plasmon field on the smooth interface.

Figures 7b and 8b illustrate the fields as they propagate towards the far zone of the slit. In the case of a slit without corrugations, the far zone is effectively reached after a propagation distance of just a few wavelengths, while in the case of the corrugated rear interface, this requires propagation over a few tens of wavelengths. In these illustrations, the entire superperiod is shown in the *x* direction to illustrate the effectiveness of the PMLs (darker bars on bottom and top) in FMM simulation of non-periodic structures: there is no visible coupling of light from neighboring superperiods near the PML layer, which (if present) would be seen as interference near the darker bars.

Finally, Figures 7c and 8c show field distributions in the focal regions of an imaging lens with NA = 1.2 and linear magnification of × 10. These results were obtained using Abbe’s theory of imaging, by retaining only those spatial frequencies of the diffracted field that fall within the NA of the collection lens. The focal fields are symmetric about the geometrical image plane at *z* = 0. Figure 8c shows clearly the formation of the focus by interference of the incoming narrow light beam and the wide pedestal arriving at larger angles within the image-space numerical aperture. In the case of the slit aperture in Figure  7c, the focal spot has only weak side lobes and is essentially diffraction limited. The corrugations increase the side lobe level considerably even at the best focus, indicating that the field immediately behind the exit plane of the probe contains strong phase variations. While the aberrations of grating-based plasmonic collimation systems are worth more careful studies, the increased side lobe level is of little concern in the present application: the area of the detector placed at the image plane can be chosen large enough to capture all side lobes with significant amplitude.

In all of the previous simulations, the incident Gaussian beam was assumed to be centered at the slit, but in the experiments, we scanned it in the *x* direction. We now proceed to simulate the effects of such scanning. First, however, it should be noted that since the slit width *w* = 50 nm is several times smaller than the 1/e half-width *W* = 200 nm used in the simulations, the incident field amplitude across the slit aperture is essentially constant. Even if this were not true, light coupling into the slit and propagation though it would make the field behind the exit plane of the probe virtually symmetric about the *z* axis. Therefore, also the field amplitude distributions in the focal region are virtually independent on the position of the incident field; only the measured intensity changes and therefore allows the profiling of the incident field without moving the detector.

**Figure 9:**
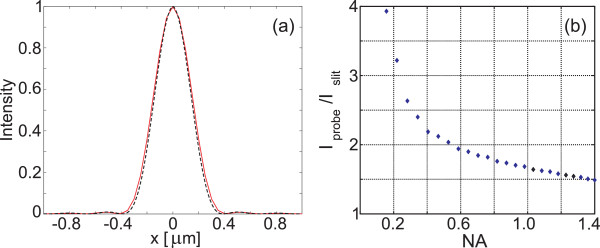
**Simulated transmittance. (a)** Magnetic field intensity of the incident beam at the entrance plane of the probe (black line) and the simulated measurement result (red line), normalized to have a unit peak value. **(b)** Dependence of the sensitivity gain factor achieved by having the corrugations in the probe, plotted as a function of the collection NA.

Figure 9a shows a comparison of the magnetic intensity profile |*H*_*y*_|^2^ of the incident field and the result of simulated measurement through the probe under conditions that approximate our experimental setup. For the convenience of resolution judgment, the peak values of both profiles have been normalized to unity, and the profiles are identical almost within the plotting precision. The simulated measured profile is slightly wider than the true incident field owing to the finite width of the slit. A normalized plot of simulated measurement without the corrugations in the probe gives a profile indistinguishable from the red curve in Figure 9a. However, the advantage of having the corrugations is obvious from Figure 9b. Here, we compare the peak values of the measured signal with and without the corrugations as a function of the numerical aperture of the collection optics. Without the corrugations, the beaming effect disappears, and hence, the sensitivity gain for small numerical apertures is as high as 3 to 4. At NA=1.4, which corresponds to our experimental setup, the theoretical gain factor is still approximately 1.5.

**Figure 10:**
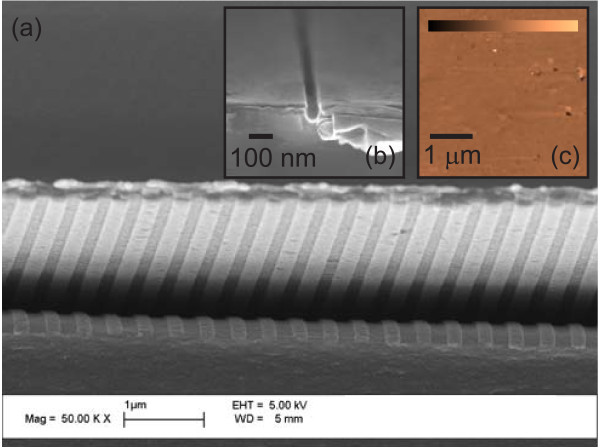
**Images of the structure. **Scanning electron microscope (SEM) and atomic force microscope (AFM) images of the structure. **(a) **SEM image of the Al glue interface, **(b) **SEM image of the entrance surface showing the slit, and **(c) **AFM image of the top surface, where the color bar indicates depth scale from -10 nm (black) to 10 nm (white).

Scanning electron micrographs of the device taken during the fabrication process are presented in Figure 10a and in the inset Figure 10b, where the grating-glue interface and the slit in the aluminum film are shown, respectively. In Figure 10a, the glue was partially peeled off from the Al layer (on the bottom of the figure) due to cutting of the structure for cross-sectional imaging, but high-accuracy penetration into the grooves is visible from the modulation. The slit shown in Figure 10b is not etched completely through; hence, a longer etch time was used to fabricate the final probe. The inset of Figure 10c shows the AFM image of the top surface without the TiO_2_ layer to illustrate the high-quality metal surface obtained by the template stripping process. Apart from a few visible pits and grains sized up to 10 nm in *z* direction, the average rms roughness of the imaged surface within the 4 *μ*m × 4 *μ*m area was only 0.5 nm. For comparative measurements, we also fabricated a probe without the corrugations.

**Figure 11:**
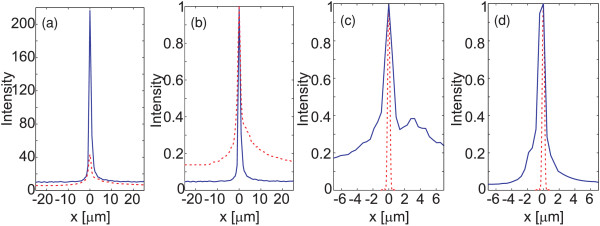
**Experimental results. (a) **Comparison of measured signals without (red) and with (blue) corrugations in the probe. **(b) **The same as the previous, but the peaks of both signals are normalized to unity. **(c) **Comparison of measured and theoretically predicted signals for the probe without corrugations. **(d) **The same as the previous but for the corrugated probe.

The signal measured by the confocal system through the probe as a function of the incident beam position is shown in Figure 11a, where we averaged the *x* profiles over 200 scan lines at different *y* positions. The red line illustrates the results obtained with the probe containing only the slit, and the black line illustrates those obtained with the corrugated probe. The enhancement by the corrugation is about fivefold, which is about three times more than the simulations for the ideal model predict in Figure 9b. In measurements with and without the corrugations, there is some background noise present even when the incident beam is positioned well outside the slit, which is at approximately the same level in both cases. In Figure 11b, both detected signals are scaled to have a unit peak intensity, showing a significant reduction in the relative background noise level when the corrugations are present. This background is most likely due to ambient room light because the probe/detection system was not fully boxed to allow only light transmitted by the slit to reach the detector. Furthermore, although the entrance Al surface is of high quality because of the stripping process, the interior of the Al film is somewhat granular, and therefore, a small fraction of the incident light may pass through the film and reach the detector.

The measured intensity profiles (averages over 40 scan lines) are compared to theoretical predictions in Figure 11c,d for samples without and with corrugations, respectively. The theoretical curves are plotted assuming that the beam waist is located at the entrance plane of the probe. However, in our setup, we had no means to ascertain this directly. Because the Rayleigh range of the focused incident beam in our setup was only approximately 200 nm, a *z*-positioning error of less than one wavelength would explain the observed broadening of the spot at, say, the half-maximum points. Additional broadening on the ‘bottom’ of the intensity profiles is also seen, making the observed profiles non-Gaussian. One possible explanation is that, as seen in Figure 10c, there are some randomly distributed imperfections in the stripped entrance surface of the Al film. Any such disturbances cause the excitation of localized surface plasmon waves, which can reach the slit and be partially transmitted by it even if the illumination spot is completely outside the slit area. Moreover, the slit wall quality, due to certain porosity of inner Al structure, may cause some perturbation to the measured signal. These effects could be avoided by further refining the template stripping and deposition processes.

Potentially, the proposed device, with a two-dimensional hole-grating structure fabricated on a protrusion, could be used as a scanning near-field optical microscope. However, we are currently limited to fabricate the probe on a flat substrate, which complicates its placing in an evanescent field above an object. Finally, we note that two-dimensionally structured arrangements containing non-symmetric and multiple tiny holes hold potential for directly measuring the local polarization and spatial coherence properties of finely structured free fields.

## Conclusions

We have proposed a scheme for direct characterization of free-space fields using a probe with a nanoaperture surrounded by periodic corrugations. The advantages of adding the corrugations to the probe were clearly demonstrated, and it is likely that the device measured the true spot size at a high accuracy. However, signal-to-noise performance of the first prototype probe still leaves much room for improvement. Besides refining the fabrication process, we believe that significant improvements in this respect can be obtained with the next-generation probe with a circular aperture surrounded by circular corrugations. Such a probe can be designed along the lines presented above and fabricated using the process introduced in this paper.

## Competing interests

The authors declare that they have no competing interests.

## Authors’ contributions

The structures were fabricated by JR, the numerical work was carried out by JR and HJH, the experimental part was performed by JR and SR, and the manuscript was written by JT, JR, HJH, and SR. All authors read and approved the final manuscript.
